# Bilateral Pulmonary Embolism in a Discharged Patient With Resolved COVID-19 Pneumonia

**DOI:** 10.7759/cureus.9406

**Published:** 2020-07-26

**Authors:** Mrunal Koche, Samuel Bechmann, Ivie S Omoruyi

**Affiliations:** 1 Emergency Department, Wyckoff Heights Medical Center, Brooklyn, USA; 2 Emergency Department, Wyckoff Heights Medical Center, Brookyln, USA

**Keywords:** covid-19, pulmonary embolism (pe), deep vein thrombosis (dvt)

## Abstract

Thromboembolic events with coronavirus disease 2019 (COVID-19) infection, such as pulmonary embolism, have been described in recent literature as a manifestation in patients during their hospital admission. Our case report describes a delayed manifestation of bilateral pulmonary embolism in a patient who was discharged home. The patient is a 40-year-old COVID-19 positive male that presented to the emergency department eight days after his discharge with shortness of breath and diaphoresis. On triage, the patient was hypoxic and tachycardic, prompting a high index of suspicion for pulmonary embolism. Computed tomographic angiography of the chest was performed confirming the presence of a bilateral pulmonary embolism. Subsequently, the patient was started on heparin and transferred to a tertiary facility for thrombectomy.

Pulmonary embolism is a manifestation of acute COVID-19 infection. It is important for clinicians to have an increased suspicion for pulmonary embolism in patients presenting with worsening dyspnea and hypoxia who were recently admitted for acute COVID-19 pneumonia. Patients that were hospitalized for acute presentation of COVID-19 infection should reasonably be considered for extended anticoagulant therapy after discharge.

## Introduction

Since the beginning of the coronavirus disease 2019 (COVID-19) pandemic, also referred to as novel SARS-CoV-2 (severe acute respiratory syndrome coronavirus-2), ongoing research has been providing information on the pathogenesis and heterogeneous presentation of the virus. One such manifestation is the development of thromboembolic events [1]. The manifestation of deep vein thrombosis (DVT) and pulmonary embolism (PE) in the acute presentation of COVID-19 pneumonia has been reported with a higher prevalence in patients admitted to the hospital, specifically the intensive care unit (ICU) [2-6]. Pulmonary embolism (PE) is a result of a dislodged clot that occludes the pulmonary circulation commonly originating from the lower extremities. The most common signs and symptoms include dyspnea, tachycardia, hypoxia, pleuritic chest pain, or cough; and less frequently patients can present with hemoptysis, orthopnea, or shock [7]. We present a patient who was hospitalized for fifteen days for acute COVID-19 pneumonia who developed a delayed manifestation of bilateral PE eight days after he was discharged home. The patient had no predisposing thromboembolic risk factors, and during the course of the hospitalization the patient was on DVT prophylaxis. To the best of our knowledge, and in review of medical literature, this is the first time there has been a case with a bilateral pulmonary embolism in a patient with resolved COVID-19 pneumonia.

## Case presentation

**Figure 1:**
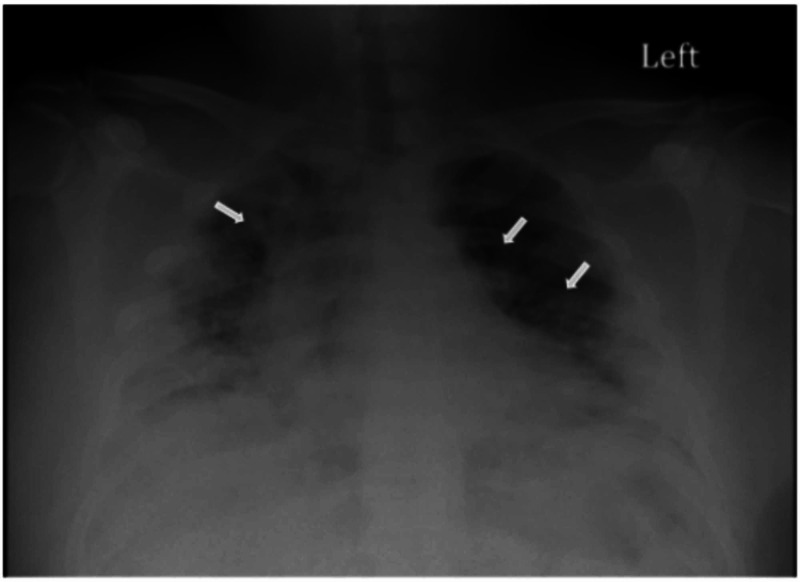
Chest X-ray taken during the patients' initial hospital visit, showing diffuse bilateral pulmonary disease (white arrows)

A 40-year-old Hispanic male with a history of hypertension and type 2 diabetes mellitus presented to the emergency department (ED) with complaints of fever, worsening cough, and shortness of breath for seven days. Initial triage vitals were 100.4^o^F (38^o^C), oxygen saturation of 85% on room air, and a heart rate of 117 beats per minute. On physical examination, the patient appeared to be in mild respiratory distress, ill-appearing and had bilateral rales on auscultation. He was immediately placed on supplemental oxygen with a nonrebreather mask (NRB). Laboratory analysis showed lymphocytopenia 8.7% (nl range 25-50%), hemoglobin levels 16.7 g/dL (13.0 - 17.0 g/dL), lactate dehydrogenase 986 U/L (84 - 246 U/L), procalcitonin 0.24 ng/mL (0.05-0.09 ng/mL), and arterial blood gas showed pH 7.46, partial pressure of carbon dioxide (pCO2) 33, partial pressure of oxygen (pO2) 47, bicarbonate (HCO_3_) 23.5 and retinal arterial oxygen saturation (sO2a) of 87%. A chest x-ray was the only imaging modality performed, which was remarkable for diffuse bilateral airspace disease (Figure 1).

The patient was given acetaminophen 975 mg, azithromycin 500 mg oral, nasopharyngeal swab for SARS-CoV-2 was performed and later was admitted to our institution for bilateral pneumonia secondary to suspected COVID-19 viral infection. Upon day 1 of admission, the patient was continued on azithromycin 250 mg oral daily for four days and was started on methylprednisolone 40 mg intravenous (IV) push every 12 hours, zinc sulfate 220 mg daily, and DVT prophylaxis (enoxaparin 40 mg subcutaneous injection daily). On admission day 2, the SARS-CoV-2 test was resulted as positive; subsequently, the patient was started on hydroxychloroquine 400 mg. However, hydroxychloroquine was discontinued after two doses due to the prolongation of QTc. 

Over the course of eight days, the patient continued to receive DVT prophylaxis and supplemental oxygen by NRB. Labs continued to improve, and the patient showed improvement in maintenance of his respiratory status with oxygen saturations ranging from 94-96%. Intermittent weaning trials from NRB to nasal cannula (NC) were successful on day 9 of admission. On admission day 10, the patient was transferred from our institution to a temporary short-term facility (Javits Center) for the continuation of supplemental NC oxygen therapy. In the remaining five days at the Javits Center, the patient continued to receive DVT prophylaxis and supplemental oxygen by NC. He continued to show clinical improvement and was subsequently discharged home with no further anticoagulation provided. 

**Figure 2:**
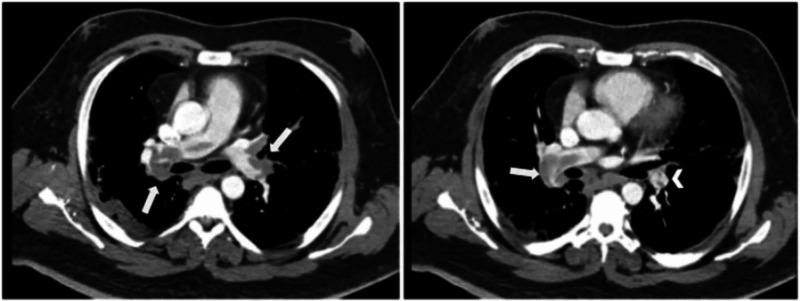
Computed tomography angiography (CTA) of the chest Axial view of the thorax showing pulmonary embolism affecting the bilateral main pulmonary arteries (white arrows). The partial filling defect surrounded by contrast material produces a polo mint sign, in keeping with acute embolus (arrowhead).

One week after the patient was discharged from the Javits Center, he returned to the ED for worsening exertional dyspnea and diaphoresis. During triage, the patient was dyspneic, hypoxic, tachycardic, and afebrile. On evaluation, the patient was visibly well-appearing, speaking in full sentences, and had an unremarkable physical examination. Laboratory analysis showed mild leukocytosis 11.30 k/uL (4.5 - 10.9 k/uL), lymphocyte 18.7% (25 - 50%), mild elevation in troponin I 0.047 ng/mL (0.015-0.04 ng/mL), normal basic coagulation profile, and an electrocardiogram (ECG) showed sinus rhythm with no acute changes. A high degree of suspicion was made for a possible PE. The Wells criteria was used for risk stratification, which was concerning for moderate risk for PE (score calculated to be 3.5). Computed tomographic angiogram (CTA) of the chest was performed, revealing a large bilateral pulmonary embolism extending into the bilateral upper and lower segmental pulmonary and subsegmental arteries (Figure 2). The patient was immediately started on intravenous heparin 80 units/kg bolus, followed by a continuous infusion of 18 units/kg/hour. Vascular surgery and the critical care team were consulted, and the decision was made to transfer the patient to a tertiary facility for thrombectomy.

The patient arrived at the ED of the tertiary center in stable condition with a heart rate of 110 bpm and oxygen saturation at 98-100% on 4L NC. ECG was performed, which showed no ischemic changes, troponin I was negative, and the pro-brain natriuretic peptide (BNP) was elevated at 3083 pg/mL. The following morning the patient underwent Inari mechanical thrombectomy with the retrieval of large clots from the right and left pulmonary arteries. On post-op day 1, the patient showed moderate improvement in hemodynamics, and his supplemental oxygen was downgraded from NC to room air with oxygen saturation maintained at > 96%. 

On post-op day two, because of the elevation in BNP and signs of right heart strain on CTA, a transthoracic echocardiogram was performed, which revealed moderate to severely decreased global left ventricular systolic dysfunction, and a left ventricular ejection fraction of 25-30%. Spectral doppler showed an impaired relaxation pattern of the left ventricular myocardial filling (grade I diastolic dysfunction), possibly related to COVID -19 myocarditis. On post-op day 3, the patient was discharged home on daily apixaban 10mg oral twice daily, followed by 5mg oral twice daily, and 2L of supplemental oxygen as needed. A two-week follow up with the patient was performed to assess the patient's clinical status. The patient reported continued improvement in clinical symptoms and reports using the supplemental oxygen less frequently.

## Discussion

There has been an increase in patients returning back to the emergency department with worsening hypoxia post-discharge who were admitted for acute COVID-19 pneumonia. With emerging data and postmortem findings of microthrombi, pulmonary emboli are being reported at an increased rate in this patient population [3-6]. SARS-CoV-2 is a novel viral infection that commonly causes pneumonia with bilateral ground-glass opacities. The heterogenic properties of COVID-19 infection have been associated with an immense inflammatory response, endothelial damage, and cytokine storm which can predispose patients to complications of disseminated intravascular coagulation, thromboembolic events, hemorrhages, and acute strokes. Other complications have included arrhythmias, acute cardiac injury, and cardiomyopathy [3, 6, 8, 9]. The common inflammatory markers observed in moderate to severe COVID-19 infections are interleukin-6 and C-reactive protein [8].

As anecdotal and research data are emerging, there is noticeable variability in the reported incidence rates of venous thromboembolism (VTE) in COVID-19 infections. The significant differences in rates may be related to the acuity of illness, along with screening and diagnostic practices for testing and confirming of VTE. The current prevalence rate of VTEs in patients who were admitted to the ICU for acute presentation of COVID-19 infection is reported to be at a range of 8.7-30% and in non-ICU patients 3-10% [10-12]. Therefore, the increase in mortality in hospitalized patients for COVID-19 infection may be associated with the development of PE, despite patients being on DVT prophylaxis during their hospital stay [4, 5].

In patients infected by SARS-CoV-2, it is extremely important to consider vascular complications as a possible cause of clinical deterioration. Overlooking thromboembolic phenomena could lead to a poor outcome and could partially explain the poor survival rates described in critically ill patients. Thus, it is reasonable to consider extended anticoagulant therapy during discharge in patients that were admitted for acute COIVD-19 pneumonia. The currently approved therapies for VTE prophylaxis for patients that were hospitalized for acute illness are [13, 14]:

- Apixaban (Eliquis®) 2.5 mg twice daily for 10-14 days but can be extended to 35 days;

- Rivaroxaban (Xarelto®) 10 mg daily for 31-39 days;

- Betrixaban (Bevyxxa®) 160 mg on day 1, followed by 80 mg once daily for 35-42 days.

## Conclusions

This case report serves as an example of the potential severity of thromboembolic complications in patients who are discharged after hospitalization for acute COVID-19 pneumonia. The overlap in the signs and symptoms of COVID-19 associated respiratory distress and concurrent pulmonary embolism creates a diagnostic challenge for physicians. As demonstrated in the case report, surge in cytokine storm and endothelial damage with the COVID-19 pneumonia can lead to deep vein thrombosis and pulmonary embolism. This case will help general practitioners, emergency medicine physicians, pulmonologists and cardiologists to formulate the differential diagnosis and request appropriate investigations. Patients that were hospitalized for acute presentation of COVID-19 infection should reasonably be consider for extended anticoagulant therapy after discharge.
